# Genetics and epigenetics of arrhythmia and heart failure

**DOI:** 10.3389/fgene.2013.00219

**Published:** 2013-10-30

**Authors:** Burcu Duygu, Ella M. Poels, Paula A. da Costa Martins

**Affiliations:** Department of Cardiology, CARIM School for Cardiovascular Diseases, Maastricht UniversityMaastricht, Netherlands

**Keywords:** arrhythmias, heart failure, genetic predisposition to disease, epigenetic regulation, microRNAs, pharmacoepigenomics

## Abstract

Heart failure (HF) is the end stage of several pathological cardiac conditions including myocardial infarction, cardiac hypertrophy and hypertension. Various molecular and cellular mechanisms are involved in the development of HF. At the molecular level, the onset of HF is associated with reprogramming of gene expression, including downregulation of the alpha-myosin heavy chain (α-MHC) gene and sarcoplasmic reticulum Ca ^2+^ ATPase genes and reactivation of specific fetal cardiac genes such as atrial natriuretic factor and brain natriuretic peptide. These deviations in gene expression result in structural and electrophysiological changes, which eventually progress to HF. Cardiac arrhythmia is caused by altered conduction properties of the heart, which may arise in response to ischemia, inflammation, fibrosis, aging or from genetic factors. Because changes in the gene transcription program may have crucial consequences as deteriorated cardiac function, understanding the molecular mechanisms involved in the process has become a priority in the field. In this context, various studies besides having identified different DNA methylation patterns in HF patients, have also focused on specific disease processes and their underlying mechanisms, also introducing new concepts such as epigenomics. This review highlights specific genetic mutations associated with the onset and progression of HF, also providing an introduction to epigenetic mechanisms such as histone modifications, DNA methylation and RNA-based modification, and highlights the relation between epigenetics, arrhythmogenesis and HF.

## INTRODUCTION

Genetic mutations can contribute to the diverse pathologies of heart failure (HF) by altering structure and therefore, the function of proteins responsible for various cellular activities (Creemers et al., 2011). While several studies have been devoted to the evaluation of genetic factors related to heart disease and genetic complications, much less is known about the relevance of epigenetics. The term “epigenetics” is defined as changes in gene expression that cannot be explained by changes in DNA sequence (Egger et al., 2004) but rather result from alterations related to packaging and/or translation of genetic information (Bird, 2007). Epigenetic mechanisms can be acquired or heritable and constitute a mean by which interactions between genes and environment can occur. Epigenetic regulation occurs by three key mechanisms: (i) methylation of CpG islands, mediated by DNA methyltransferases (DNMTs), (ii) modification of histone proteins and (iii) microRNAs (miRNAs). Such modifications will lead to differential expression of similar information depending on the surrounding conditions, resulting in gene activation or silencing. Although epigenetic variability of genetic information is part of normal development and differentiation, it also depends on exogenous stimuli (e.g., smoking, drug abuse) and can, therefore, reflect the influence of those factors on the development of disease (Feinberg, 2007). The role of epigenetics has been mainly evaluated in cancer but recent studies have begun to address the involvement of epigenetics in the development and progression of cardiovascular diseases (CVD).

Heart failure is the end stage of several pathological cardiac conditions including myocardial infarction (MI), cardiac hypertrophy and hypertension. Various molecular and cellular mechanisms are involved in the development of HF. At the molecular level, the onset of HF is associated with reprogramming of gene expression, including downregulation of the alpha-myosin heavy chain (α-MHC) gene and sarcoplasmic reticulum Ca^2+^ ATPase genes and reactivation of specific fetal cardiac genes such as atrial natriuretic factor (ANF) and brain natriuretic peptide (BNP; Herron and McDonald, 2002; Olson et al., 2006). These deviations in gene expression result in structural and electrophysiological changes, which eventually progress to HF. Cardiac arrhythmia is caused by altered conduction properties of the heart, which may arise in response to ischemia, inflammation, fibrosis, aging or from genetic factors. Because changes in the gene transcription program may have crucial consequences such as deteriorated cardiac function, understanding the molecular mechanisms involved in the process has become a priority in the field. In this context, various studies besides having identified different DNA methylation patterns in HF patients (Movassagh et al., 2011a, 2011b), have also focused on specific disease processes (Yan et al., 2010; Kim et al., 2011) and their underlying mechanisms (Baccarelli and Bollati, 2009; Handy et al., 2011; Shirodkar and Marsden, 2011), also introducing new concepts such as epigenomics. This review highlights specific genetic mutations associated to the onset and progression of HF, also providing an introduction to epigenetic mechanisms such as histone modifications, DNA methylation and RNA-based modification, and highlights the relation between epigenetics, arrhythmogenesis and HF.

## GENETICS OF HEART FAILURE

Genetic forms of HF are mainly known as familial dilated cardiomyopathy (FDCM). There are, however, two other familial forms of cardiomyopathy: hypertrophic cardiomyopathy (FHCM) and arrhythmogenic right ventricular cardiomyopathy (ARVC). In fact, FHCM is the most common form of inherited HF with a prevalence of 1 in every 500 individuals (Rodriguez et al., 2009). FHCM is mainly defined as unexplained left ventricular hypertrophy with increased heart mass (Elliott and McKenna, 2004). The majority of patients with FHCM (approximately 60%) exhibit autosomal dominant mutations in genes encoding for sarcomere proteins such as β-myosin heavy chain (MYH7), cardiac myosin binding protein C (MYBPC3), cardiac troponin T (TNNT2), troponin I (TNNI3), alpha-tropomyosin (TPM1), myosin light chains (MYL2 and MYL3) and cardiac actin (ACTCI; Morita et al., 2008, 2010; Lopes and Elliott, 2013).

Familial dilated cardiomyopathy is characterized as idiopathic DCM with a prevalence of 20–50% determined by epidemiological studies using family history and clinical, electrocardiographic and echocardiographic screening of first-degree relatives (Michels et al., 1992; Grunig et al., 1998). FDCM is mainly inherited in an autosomal dominant manner (approximately 90%) however, X-linked (5–10%) and much less commonly autosomal recessive (AR) or mitochondrial inheritance have also been reported (Hershberger et al., 2009). A genetic cause of FDCM was identified in 30–35% cases and mainly mutant variants of Laminin A/C (LMNA) have been reported as the most common cause of FDCM (in 7.3% of patients with DCM; Hershberger et al., 2009; Hershberger and Siegfried, 2011). In a recent study, Titin (TTN) truncating mutations were attributed as the cause of FDCM in 27% of a total of 312 DCM patients (Herman et al., 2012). Furthermore, GATA zinc finger domain containing protein 1 (GATAD1) has been identified as a disease-causing gene for AR DCM by genome-wide mapping and exome sequencing in a unique family (Theis et al., 2011).

## EPIGENETIC MECHANISMS

There are several epigenetic mechanisms in eukaryotes and many have already been linked to cardiac development, CVD and/or HF. The main alterations encompassing epigenetics in CVD are described below and include ATP-dependent chromatin remodeling, DNA methylation, histone modification and RNA-based mechanisms.

### CHROMATIN REMODELING THROUGH ATP-DEPENDENT ENZYMES

The ATP-dependent chromatin-remodeling complexes do not perform covalent modifications of the DNA or histones but rather use energy derived from ATP hydrolysis to move, destabilize, eject or restructure nucleosomes. There are four different families of ATP-dependent chromatin remodeling complexes: switching defective/sucrose non-fermenting complexes (SWI/SNF); imitation switch complexes (ISWI); chromodomain, helicase, DNA binding complexes (CHD) and inositol-requiring 80 complexes (INO80; Li, 2002; Yang and Seto, 2008; Clapier and Cairns, 2009; Ho and Crabtree, 2010). Although all members of each family have distinct flanking domains, they all share an evolutionarily conserved SWI-like ATPase catalytic domain that serves as vehicle to adjust histone-DNA contacts for DNA movement and chromatin restructuring. In turn, the other domains act in the recognition of covalently modified histones, modulation of ATPase activity and/or interaction with other chromatin and transcription factors. Consequently, these unique domains and their associated proteins determine the genomic targeting specificity and biological functions of each family of chromatin remodelers. In fact, chromatin modification through ATP-dependent enzymes is associated with regulation of expression of distinct gene programs in organ development and adaptation (Ho and Crabtree, 2010).

### DNA METHYLATION

**FIGURE 1:**
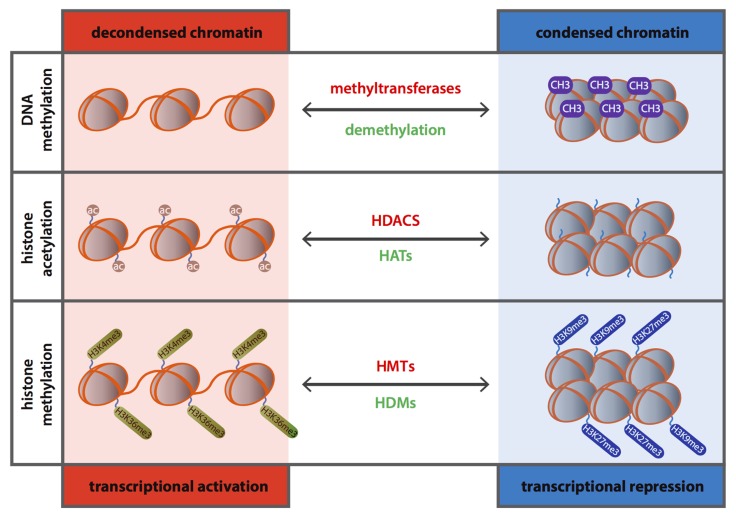
Schematic representation of the role of DNA methylation and histone modifications in transcriptional gene regulation.

DNA methylation is the most common epigenetic modification in the mammalian genome. This long-term stable epigenetic modulation involves the addition of a methyl group to the 5′ carbon of a cytosine by DNMT enzymes (**Figure 1**) and mostly occurs at the CpG (cytosine preceding guanosine) dinucleotide sequences, also known as CpG islands, in the mammalian genome (Feinberg, 2008). CpG islands, in contrast to the remainder genome, are cytosine-guanosine-rich sequences (CpG-rich), generally not methylated (Deaton and Bird, 2011), and mostly acting as sites of transcription initiation once they are associated with promoter regions of genes (~70% of gene promoters; Li et al., 1993; Saxonov et al., 2006). DNA methylation is known to be catalyzed by three different DNMTs: DNMT1, DNMT3a and DNMT3b (Broadbent et al., 2008), where DNMT1 is the core enzyme in mammals. Methylation of DNA is considered a maintenance function of DNMTs as it results in post-replicative restoration of hemi-methylated sites to full methylation (Laird, 2003). Reduction of DNMT1 activity may result in demethylation and recent studies even showed that this is an active process (Bhutani et al., 2011). However this has not been shown yet for the cardiovascular system.

DNA methylation is, generally, attributed to gene silencing by hampering the accessibility of *cis*-DNA binding elements present in the promoter regions of genes of the transcriptional machinery (Suzuki and Bird, 2008) and plays a crucial role in the regulation of chromatin structure including X chromosome inactivation, genomic imprinting, silencing of repetitive DNA elements and transposon transcription (Li et al., 1993; Panning and Jaenisch, 1996; Li, 2002). Moreover, DNA methylation has been linked to biological processes underlying various diseases from cancer (Feinberg and Tycko, 2004) to CVD, such as hypertension (Millis, 2011), diabetes (Ling and Groop, 2009; MacFarlane et al., 2009), atherosclerosis and inflammation (Wierda et al., 2010).

### HISTONE MODIFICATIONS

The eukaryotic DNA is tightly compact and organized in chromatin. The nucleosome is the central unit of chromatin and is composed of an octomer center of two copies of each histone protein (H2A, H2B, H3, and H4; Jenuwein and Allis, 2001) around which a DNA segment of 14–150 base pairs is looped. Each histone has an amino-terminal tail that protrudes from the surface of the nucleosome and which can be subjected to various posttranscriptional modifications such as phosphorylation, sumoylation, ubiquitination, methylation, ADP-ribosylation, proline isomerization, deimination and acetylation (Handy et al., 2011). These modifications lead to conformational changes in the chromatin resulting in altered gene expression (Margueron and Reinberg, 2010) depending on whether DNA becomes accessible (euchromatin) or inaccessible (heterochromatin) for transcription (**Figure 1**).

### HISTONE ACETYLATION

Histone acetylation occurs at the lysine residues of the histone tails resulting in de-condensation of the chromatin structure and serving as a binding site for bromodomain proteins and transcriptional activators, and eventually leading to transcriptional activation (Elliott and McKenna, 2004; Rodriguez et al., 2009). Conversely, histone deacetylation induces chromatin condensation and therefore transcriptional repression (Clayton et al., 2006; Shahbazian and Grunstein, 2007; **Figure 1**). Acetylation of histones is a dynamic process mediated by two counteracting enzyme families, the histone acetyltransferases (HATs) and histone deacetylases (HDACs). The harmony between the activities of these two sets of enzymes is a crucial element during regulation of gene expression and its deregulation is linked to several pathological conditions varying from cancer to CVD (Ordovas and Smith, 2010; Burgess, 2012).

### HISTONE METHYLATION

Other key modulator of posttranslational regulation is histone methylation which can occur on all basic amino acid residues of the histone tail; arginines, lysine and histidines (Cheung and Lau, 2005). In addition, different amino acids can be methylated to a different extent and while lysine can be subjected to mono-, di- and trimethylation, arginine residues can only become mono- or dimethylated (Cheung and Lau, 2005). Methylation of histones is a dynamic process mediated by histone methyltransferases (HMTs) and histone demethylases (HDMs; Teperino et al., 2010) and, unlike acetylation, histone methylation can induce either activation or repression of gene expression depending on the target sites and degree of methylation (Lachner and Jenuwein, 2002; **Figure 1**). In contrast to histone acetylation, histone methylation governed mainly by HMTs SUV39H1 and G9a (Martin and Zhang, 2005; Shi and Whetstine, 2007), has long been considered to be a permanent epigenetic mark (Jenuwein and Allis, 2001). However, the discovery of new players such as HDMs has shifted the paradigm and, in fact, several studies showed that histone methylation is tightly regulated in inflammatory and metabolic disorders (Saccani and Natoli, 2002; Villeneuve et al., 2008; Brasacchio et al., 2009).

### RNA-BASED MECHANISMS

It is now proven and accepted that the majority of the genomic DNA is transcribed as non-coding RNAs and that such RNA species play pivotal regulatory roles during development (Sayed and Abdellatif, 2011), in response to environmental adversity (Ferguson, 2011), and at the onset and progression of disease (Sayed and Abdellatif, 2011). In this context, many studies were directed at revealing the role of non-coding RNAs in physiological and pathological processes.

There are two main classes of non-coding RNAs: infrastructural (small nuclear and nucleolar RNAs, ribosomal RNAs) and regulatory RNAs (miRNAs, long non-coding RNAs, small interfering RNAs and Piwi-interacting RNAs). To date, only miRNAs have been associated with epigenetic regulatory mechanisms in HF. Epigenetic regulation through long non-coding RNAs have been extensively studied in cancer but have also been associated with cardiovascular disease, mainly in maintenance of vascular homeostasis (Robb et al., 2004; Li et al., 2010).

### MICRORNAs

MicroRNAs were first described in the nematode *Caenorhabditis elegans*, in the early 1990s (Lee et al., 1993). From then on, a multitude of miRNAs have been identified and investigated, and presently there are ~1600 human miRNA sequences annotated at miRBase19 (Kozomara and Griffiths-Jones, 2011).

MiRNAs are transcribed as primary transcripts (pri-miRNA) from intergenic, intronic or exonic regions in the genome, by RNA polymerase II. These pri-miRNAs fold into an hairpin shape with a five prime (5′) capped (mGpppG) and a polyadenylated tail which is subsequently cleaved by an enzyme complex composed of the RNase III endonuclease Drosha and the dsRNA binding protein Pasha (also known as DiGeorge critical region 8; DGCR8; Lee et al., 2003, 2004). The resulting shorter (70–100 nucleotide in length) hairpin-shaped precursor miRNA (pre-miRNA) is transported from the nucleus into the cytoplasm by Ran-GTP and exportin-5 (Kim, 2004). In the cytoplasm, pre-miRNAs are further processed by a RNase III enzyme, Dicer, into a short (20–25 nucleotides in length) transient double stranded RNA molecule. At this stage, the formed mature RNA molecule is included in a protein complex – the so-called RNA-inducing silencing complex (RISC), while the passenger strand is degraded (Winter et al., 2009). The RISC-miRNA complex specifically targets mRNA sequences leading to negative regulation of protein synthesis or mRNA degradation (Winter et al., 2009). One miRNA can regulate a vast number of mRNAs simultaneously (Lewis et al., 2005) by predominantly acting through destabilization of target mRNAs and subsequently leading to reduced protein output (Guo et al., 2010). Therefore, decreased protein production can result from a combination of mRNA destabilization and translational inhibition. MiRNAs have been shown to be involved in different pathological processes such as cancer and CVD (Lujambio and Lowe, 2012; Quiat and Olson, 2013). While in cancer epigenetic mechanisms have been widely associated with silencing of miRNA-encoding genes and thus recognized to greatly influence the expression of genetic information, only recently the importance of such mechanisms have started to be addressed in CVD, and more specifically in HF.

## EPIGENETICS AND ARRHYTHMIA

Recent technological advances in DNA sequencing have enabled epigenome mapping and provided unprecedented insight into the distribution, interplay, and potential novel functions of chromatin modification and associated proteins. Remarkably, when using such technologies in evaluating the heart rhythm prominence of selected gene networks including epigenetic modulators, not previously associated with arrhythmia, were identified as relevant under particular circumstances. A first evidence for epigenetic regulation of cardiac rhythm was raised from a study conducting microarrays on heart rhythm determinants (HRD) on tissue from mice exposed to either intermittent or chronic hypoxia and untreated wild type mice. A different environment (hypoxia) profoundly restructured the HRD web by changing the hierarchy of the composing genes and by identifying new role players. This was the case for the epigenetic modulators HDAC5, Mef2b and Mef2c (Iacobas et al., 2010).

### CHROMATIN REMODELING AND ARRHYTHMIA

Postural tachycardia syndrome (POTS) has multiple symptoms, one of such being tachycardia. Dysfunction of the norepinephrine transporter (NET) gene has previously been implicated in POTS, with a reported coding mutation in the NET gene (SLC6A2; Bayles et al., 2012). Head-up tilt experiments in POTS patients and showed that the expression of norepinephrine transported is lower in POTS patients compared to healthy subjects. In the absence of altered SLC6A2 gene sequence or promoter methylation, the observed reduced expression of norepinephrine was directly correlated with chromatin modifications. Changes in expression were attributable to increased binding of the repressive methyl CpG-binding protein 2 (MeCP2) regulatory complex, in association with an altered histone modification composition at the promoter region of the SLC6A2 gene (Bayles et al., 2012).

### DNA METHYLATION AND ARRHYTHMIA

The KCNQ1 gene is located on chromosome 11 in a region that contains a cluster of 6 genes that are expressed from either only the maternal or the paternal allele. In mice, the KCNQ1 overlapping transcript (KCNQ1ot1) is transcribed from a promoter located in intron 10 of the KCNQ1 gene. This promoter region is a CpG island and undergoes methylation on the maternal chromosome, preventing transcription, and therefore allowing expression of the gene cluster. However, this promoter region is not methylated on the paternal chromosome allowing expression of the regulatory transcript and suppressing the expression of the gene cluster (Mancini-DiNardo et al., 2003). The maternal allele is transcribed in early embryogenesis with the paternal allele being progressively methylated and therefore only activated during late embryogenesis.

Variable imprinting of the KCNQ1 gene provides a possible explanation for the existence of long QT syndrome (LQTS) in the absence of a coding sequence mutation in KCNQ1. Paternal imprinting is probably relieved in cardiac tissue, meaning that during differentiation methylation of the paternal chromosome must occur to block production of the suppressive KCNQot1 transcript. Mutations that disrupt the CpG island could prevent methylation and silence the paternal allele in the heart (Mancini-DiNardo et al., 2003; Bokil et al., 2010). A more recent study by Fatima et al. (2012) associates epigenetic modifications with regulation of the ATP sensitive potassium channel (KATP). In cardiac myocytes, different isoform combinations of the SURx (SUR1, SUR2) and Kir6.2 (KCNJ11) will be responsible for distinct physiological and pharmacological properties, depending on the isoforms expressed. Promotor CpG methylation appears to be one of the regulators of SURx isoform expression and therefore, regulated or aberrant CpG methylation might play a role in controlling channel structure and function under different conditions (Fatima et al., 2012).

## HISTONE MODIFICATIONS IN ARRHYTHMIA

Histone deacetylases-1 and -2 have important functions in regulating cardiac gene expression and cardiomyocyte differentiation. While myocardium-specific deletion of either HDAC-1 or HDAC-2 results in no apparent cardiac phenotype, specific deletion of both in the murine myocardium, results in death within 2 weeks after birth, due to cardiac arrhythmias and dilated cardiomyopathy (Montgomery et al., 2007). This is likely caused by upregulation of genes that encode for fetal calcium channels and skeletal muscle-specific contractile proteins, including hyperpolarization-activated non-selection cation current (If) and T-type Ca^2+^ current (ICa, T), both involved in calcium handling. Such genes are normally transcriptionally repressed by the RE1-silencing transcription factor (REST) through class I and IIa HDACs. Knockout of both HDAC-1 and -2 seems to result in incapacity of REST to repress these fetal genes, resulting in, among other things, arrhythmia (Montgomery et al., 2007; Chang and Bruneau, 2012).

Ablation of PAX-interacting protein 1 (PTIP), a key component of the histone H3 lysine 4 (H3K4me) methyltransferase complex, leads to reduced H3K4me expression levels and is sufficient to alter subsequent gene expression profiles. One of those H3K4me-regulated genes is the Kv channel-interacting protein 2 (Kcnip2), a regulator of cardiac repolarization current that is known to have functions in arrhythmogenesis. Regulation of Kcnip2 by H3K4me leads to decreased sodium current and action potential upstroke velocity and significantly prolonged action potential duration (APD), thereby increasing the risk of lethal ventricular arrhythmias. These results suggest that maintaining H3K4me marks is necessary for the stability of a specific transcriptional program and cellular homeostasis (Stein et al., 2011; Chang and Bruneau, 2012).

In Duchenne muscular dystrophy (DMD) more than 30% of deaths result from a progressive deterioration in cardiac function. Ventricular arrhythmia is a common complication in DMD patients and a risk factor for sudden cardiac death. Colussi et al. (2010) used X-chromosome-linked muscular dystrophy (mdx) mice, a model for DMD, and treated them with the histone deactylase inhibitor suberoylanilide hydroxamic acid (SAHA). In resting state there was no difference between treated and untreated groups, however, upon restraint, an increase was seen in ventricular arrhythmias in untreated mdx animals compared to mdx SAHA- treated animals or wild type control animals. Epicardial recordings revealed prolongation of the QRS complex in mdx- untreated mice in comparison to mdx-SAHA treated mice and WT mice, together with a significant reduction in impulse propagation velocity. Further analysis revealed that SAHA induces connexin 40 (Cx40), Cx37 and Cx32 remodeling but expression of Cx43 and Cx45 remains unaltered. Treatment with SAHA not only reversed Cx43 lateralization, which was observed in mdx- untreated animals, but also re-induced Na_v_1.5 expression. This indicates that in mdx mice SAHA attenuates arrhythmias by mechanisms in which connexin-remodeling and sodium channel re-expression may play a role (Colussi et al., 2010).

Atrial fibrillation (AF), induced by atrial fibrosis, seems to also be epigenetically regulated and this was suggested in a study sought to determine whether the HDAC inhibitor trichostatin A (TSA) reduces the amount of atrial fibrosis and concomitant AF (Liu et al., 2008). Transgenic mice overexpressing the homeo-domain-only protein (HopX-Tg), which recruits HDAC activity to induce cardiac hypertrophy were either treated or untreated with TSA and compared to control groups. Invasive electrical stimulation induced more atrial arrhythmias in HopX-Tg untreated mice than in HopX-Tg TSA-treated mice. TSA reduced atrial arrhythmia duration and atrial fibrosis in HopX-Tg animals. In the HopX-Tg untreated mice, atrial Cx40 was found to be lower than in WT mice, a phenomenon that was abrogated by introducing TSA in these mice. Myocardial angiotensin II levels were similar between groups, suggesting that HDAC-inhibition reverses atrial fibrosis, Cx40 remodeling and atrial arrhythmia vulnerability, rendering the atrium almost refractory to arrhythmia inducibility, independent of angiotensin II in cardiac hypertrophy (Liu et al., 2008).

### NON-CODING RNA IN ARRHYTHMIAS

Several studies have been conducted to look at the association between miRNAs and arrhythmias. MiRNA expression profiles were shown to differ in right atrial disease, with 47 miRNAs being differentially expressed between disease and control states, whereas similar changes in expression could not be found in left atrial disease (Cooley et al., 2012; Kim, 2013). In a different study, miRNAs that were differentially expressed between AF and sinus rhythm in patients with mitral stenosis were showed by microarrays (Xiao et al., 2011; Kim, 2013). These data indicate that miRNAs play a role in regulating cardiac conduction and in the induction of arrhythmias.

Multiple studies have shown that miR-208a plays an important role in action potential conduction. Overexpression of miR-208a leads to arrhythmia, cardiac fibrosis and hypertrophy, and is a strong predictor of cardiac death (Oliveira-Carvalho et al., 2013). Genetic deletion of miR-208a, on the other hand, also leads to an increased risk of AF and other arrhythmias, due to aberrant conduction mainly caused by dysregulation of Cx40 (Callis et al., 2009; Oliveira-Carvalho et al., 2013). Similarly, also miR-328 is upregulated not only in animal models of AF but also in human tissue samples from AF patients. Overexpression of miR-328 in mice increased vulnerability to AF as confirmed by diminished L-type Ca^2+^ current and shortened atrial APD. AF vulnerability could be reversed by concomitant inhibition of the miRNA by an antagomir (Lu et al., 2010; Kim, 2013).

The most well established cardiac conduction-related miRNA is by far, miR-1. This miRNA plays a role in myotonic dystrophy, a disease where degeneration of the conduction system occurs and increased CACNA1C (CAV 1.2) expression, a cardiac L-type Ca^2+^ channel gene, is observed (Kim, 2013). The involvement of miR-1 in electrocardiophysiology was further confirmed by a targeted deletion of miR-1-2 by Zhao et al. (2007), which lead to a high rate of sudden death, caused by conduction blockade due to direct targeting of Irx5, a transcription factor that regulates cardiac repolarization. In rats, induction of MI by occlusion of the left anterior descending artery results in miR-1 upregulation and arrhythmia exacerbation but treating the animals with an antisense inhibitor could abrogate these effects. Furthermore, miR-1 also directly targets KCNJ2, which encodes for the calcium channel subunit Kir 2.1, providing a possible mechanism for increase of arrhythmias in MI (Yang et al., 2007). The role of miR-1 in arrhythmogenesis was further confirmed in humans where atrial cells from AF patients display a 86% decrease in miR-1expression, a subsequent increased Kir 2.1 protein expression and an increase in I_k1_ density (Girmatsion et al., 2009; Kim, 2013). MiR-1 is also involved in cardiac electrical remodeling in viral myocarditis where it is upregulated, resulting in decreased Cx43, which is required for transfer of electric activation, and indicating that miR-1 plays a role in intercellular communication.

Another prominent miRNA in the regulation of cardiac conduction is miR-133. Matkovich et al. (2010) showed that an increase in miR-133a leads to prolonged QT intervals. This miRNA is highly and preferentially expressed in cardiac and skeletal muscle and is known to regulate cardiac ion channels such as Kv4- encoded I_to,f_ (Kcnip2; Matkovich et al., 2010; Kim, 2013). Furthermore, the catalytic and regulatory subunits of protein phosphatase 2A (PP2A) are decreased in cardiomyocytes in chronic HF and were shown to be targets of both miR-1 and miR-133. Because pharmacologic inhibition of PP2A leads to altered diastolic Ca^2+^ waves this indicates a role for these two miRNAs in calcium handling (Belevych et al., 2011; Kim, 2013).

Interestingly, a relation between nicotine abuse and cardiac arrhythmias has been suggested by several studies. Nicotine treatment of canine atrial fibroblasts, resulted in upregulation of transforming growth factor beta-1 (TGF-β1) and TGF- beta receptor type II levels (TGF-βRII), with concomitant decreased levels of miR-133 and miR-590, both directly targeting TGF-β1 and TGF-βRII. This effect was abolished by synthetic downregulation of both miRNAs (Shan et al., 2009; Kim, 2013).

Apart from miR-1 and miR-133, there are several other miRNAs that have been associated with regulation of cardiac conduction to some extent. This is the case for miR-212 that seems to regulate inward rectifier K^+^ current density by targeting Kir 2.1 (Kim, 2013), and for miR-21 which is increased in the left atria of patients with AF and which abrogation leads to repression of atrial fibrosis and AF (Adam et al., 2012; Cardin et al., 2012; Kim, 2013). Furthermore, conditional overexpression of miR-17-92 in cardiac and smooth muscle tissue results in both dilated, HCM as well as in arrhythmias. An increase in atrial and ventricular ectopy, as well as increased susceptibility to arrhythmia was observed in homozygous and heterozygous animals. After programmed electrical stimulation all transgenic animals developed sustained and lethal ventricular tachycardia (VT) or ventricular fibrillation (VF) and these effects were mainly caused by dysregulation of two downstream targets of miR-17-92, the lipid phosphatase and tensin homolog PTEN and Cx43 (Danielson et al., 2013). Likewise, also miR-155 and miR-181 have been associated with cardiac conduction defects. Circulating levels of miR-155 are upregulated in patients with specific angiotensin receptor type 1 (AT1R) polymorphisms that have been shown to be associated with an increased risk of ventricular tachyarrhythmias and sudden death (Blanco et al., 2012). In turn, miR-181a seems to play a role in VT after MI (Li et al., 2009). Altogether, the data available regarding the relation between miRNAs and arrhythmias establish miRNAs as crucial players in regulating cardiac electrophysiology and electric potential conduction through an array of different mechanisms.

## EPIGENETIC CONTROL OF HEART FAILURE

Recent genetic and biochemical studies indicate that epigenetic changes play a crucial role in the development of cardiac hypertrophy and HF, with dysregulation in histone acetylation status being directly linked to impaired contraction of cardiac myocytes. In fact, it has been shown that there is a cardiac chamber – specific histone acetylation pattern suggesting that cardiac ventricular chambers are epigenetically distinct (Mathiyalagan et al., 2010).

### ATP-DEPENDENT ENZYMES AND CHROMATIN REMODELING IN HF

ATP-dependent chromatin remodeling complexes play crucial roles in vertebrates, mainly in organ development and adaptation. Most of them have been associated with heart development and only a few were implicated in heart disease. The BAF (brahma-associated factor) complex is the vertebrate homolog of the yeast SWI/SNF family of chromatin remodelers. In mammals, this complex contains 12 protein components from which an ATPase subunit encoded by either *Brm* (brahma) or *Brg1* (brahma-related gene 1). These two subunits, although highly homologous, exhibit distinctive functions in vivo. While several studies have demonstrated that individual subunits of the BAF complex are essential during heart development (Lickert et al., 2004; Takeuchi et al., 2007; Takeuchi and Bruneau, 2009) and may be implicated in human congenital diseases (Kitagawa et al., 2003; Bajpai et al., 2010), BRG1 was recently involved in cardiac disease (Hang et al., 2010). In embryos, *Brg1* promotes myocyte proliferation and it preserves fetal cardiac differentiation by interacting with HDACs and poly (ADP ribose) polymerase (PARP) to repress α-MHC to β-MHC shift. *Brg1* (also known as *Smarca4*) is not expressed in the adult heart but it is reactivated by stress conditions such as pressure overload. Once reactivated, Brg1 forms a complex with its embryonic partners (HDAC and PARP), to induce the pathologic α-MHC to β-MHC shift. Adult myocardial gene deletion of Brg1 inhibited cardiac hypertrophic growth and reversed the MHC switch. Accordingly, Brg1 is activated in patients with HCM, correlating with disease severity and MHC changes (Hang et al., 2010). PPAR is a nuclear enzyme known to respond to DNA damage and facilitate repair. Besides DNA repair, PPAR-1 also modulates chromatin to control the transcriptional machinery in response to diverse stimuli. Such stimuli induce PPAR activation and PAR-dependent striping of histones from chromatin, thereby favoring the opening of chromatin to allow transcriptional regulation (Tulin and Spradling, 2003; Kim et al., 2004). PARP is activated in cardiac hypertrophy and its activity is increased in murine and human failing hearts (Pillai et al., 2005b; Xiao et al., 2005). Deletion of PARP-1 in mice or pharmacological inhibition of PARP activity decreases cardiac hypertrophy induced by angiotensin II (Pillai et al., 2006) or pressure overload (Pillai et al., 2005a; Xiao et al., 2005), delays the progression from hypertensive cardiomyopathy to HF (Bartha et al., 2009), decreases cell death and HF after MI (Palfi et al., 2006) and diminishes myocardial ischemia/reperfusion injury (Szabo et al., 2002).

Although very preliminary, there seems to be a link, at the chromatin level, between fetal hearts and adult diseased hearts, and in the future, targeting the regulation of chromatin remodeling processes may become a promising approach to prevent or maybe even reverse pathological cardiac hypertrophic growth and HF.

### DNA METHYLATION IN HEART FAILURE

Unlike in other diseases such as cancer, the role of DNA methylation in HF remains elusive. Movassagh et al. (2010) compared genome-wide methylation profiles of left ventricle tissue from HF patients and healthy controls by methylated DNA immunoprecipitation-chip (MeDIP-chip), in which immunoprecipitation of methylated DNA is followed by microarray hybridization and further validated by bisulfite sequencing. As a result, three differentially methylated angiogenesis-related loci have been identified and correlated to differential expression levels of the corresponding gene (Movassagh et al., 2010). Hyper-methylation of the 5′ regulatory region of platelet endothelial cell adhesion molecule 1 (PECAM-1) and hypo-methylation of the angiomotin-like protein 2 (AMOTL2) in failing hearts correlated with reduced expression of those genes, while hyper-methylation within the Rho GTPase activating protein 24 gene (ARHGAP24) is correlated with increased expression of ARHGAP24 in failing hearts (Movassagh et al., 2010). Moreover, a follow up study (Movassagh et al., 2011a) generated a genome-wide DNA methylation map of human hearts and revealed a significant decrease in global promoter methylation of genes with increased expression in failing hearts (Movassagh et al., 2011a). The genome-wide methylation profile of patients with idiopathic dilated cardiomyopathy was recently generated (Haas et al., 2013). In an attempt to validate the methylation profiling of the 20 most differentially methylated genes, MassARRAY and Bisulfite sequencing were used in a large independent cohort (30 patients; Baccarelli and Bollati, 2009). Interestingly, 12 (out of 20) genes showed similar methylation patterns between the two independent studies. Such approach allowed the identification of two novel genes with differential methylation profiles between patient and control subjects, lymphocyte antigen 75 (ly75) and adenosine A2a receptor (adora2a). Curiously, downregulation of those genes in zebrafish by using specific morpholino technology resulted in reduced ventricular contractility and HF (Haas et al., 2013). More recently, DNA methylation was found to be responsible for the hypermutability of distinct cardiac genes. This is the case for the cardiac isoform of the myosin binding protein C gene (Mybpc3) that has a significantly higher level of exonic methylation of CpG sites than the skeletal isoform (Mybpc2; Meurs and Kuan, 2011). This suggests that there are unique aspects of the Mybpc3 gene or its epigenetic environment that are prone to generate genetic mutations.

Very recently, a report in the Journal of the American Heart Association (Bellavia et al., 2013) provided evidence for the effects of ambient particulate-matter (PM) on blood pressure (BP). In humans, exposure to fine and coarse concentrated ambient particles (CAPs) induce blood hypomethylation of *Alu*, a transposable repeated element, and Toll-like receptor 4 (TLR4). Hypomethylation of both factors was found to be associated with increased systolic BP after exposure. This is of great interest since many epidemiological studies (O𠆝Toole et al., 2008; Brook et al., 2010) have reported a correlation between PM exposure, cardiovascular disease and death, and may, therefore, represent a novel mechanism that mediates environmental effects on BP and indirectly cardiovascular disease and HF.

### HISTONE MODIFICATION IN HEART FAILURE

#### Histone acetylation

Cardiac hypertrophy is the initial response to cardiac stress leading to adverse cardiac remodeling and eventually to HF. In order to elucidate the underlying mechanisms behind the development of cardiac hypertrophy, the role of histone acetylation/deacetylation has been extensively studied. Gusterson et al. (2003) and Morita et al. (2010) demonstrated that overexpression of the transcriptional co-activators CREB binding protein (CRB) or p300, individually, could induce hypertrophic growth of cardiomyocytes depending on their histone HAT activity. Consequently, inhibition of these co-activators repressed phenylephrine (PE)-induced hypertrophic cell growth (Gusterson et al., 2003). High expression and induced activity of HAT were observed in animals subjected to pressure overload, compared to sham operated animals, while heterozygous p300 transgenic animals revealed limited cardiac hypertrophy with preserved cardiac function when subjected to pressure overload (Morita et al., 2010). Intriguingly, another study showed that p300 transgenic animals develop HF at baseline, as indicated by high mortality, adverse remodeling and impaired cardiac function (Yanazume et al., 2003). Although these studies indicate that p300 is a crucial modulator of cardiac remodeling they do not specifically address the importance of its HAT activity in vivo. To assess this question, studies with transgenic animals carrying a mutant form of p300, with no HAT activity, were performed revealing a rescue of MI-induced pathological remodeling as well as preserved cardiac function compared to intact p300-carrying transgenic animals (Miyamoto et al., 2006). These responses to p300 modulation in vivo are, most likely, related to the fact that p300 can directly acetylate non-histone proteins such as hypertrophy-responsive transcriptional factors like MEF2 (Wei et al., 2008) and GATA-4 (Yanazume et al., 2003; Miyamoto et al., 2006).

The regulation of gene expression by HDACs seems to be more complex. HDACs are divided into four different classes (class-I, -IIa, -IIb and -IV) based on differences in their structure, enzymatic function, expression patterns and subcellular localization. Class I HDACs (HDAC1, 2, 3 and 8) are expressed ubiquitously and predominantly localized in the nucleus. Class IIa HDACs (HDAC4, 5, 7, and 9) shuttle between the nucleus and the cytoplasm and are strictly expressed in muscle, heart and brain tissues (Haberland et al., 2009). A first demonstration of the relevant role of HDAC activity in cardiomyocytes derived from a study where myocardial differentiation of monkey ES cells was facilitated by TSA, an HDAC inhibitor (Hosseinkhani et al., 2007). Furthermore, differential chromatin scanning (DCS) is a technique used to genome-widely profile HDAC targets enabling the isolation of genomic fragments associated with histones and, therefore, carrying different acetylation marks (Kaneda et al., 2005). Such studies provide a basis for all following studies into the role of epigenetic modifications in cardiac disorders (**Table 1**). Interestingly, the two classes of HDACs show opposite roles in cardiac hypertrophy with class I HDACs being pro-hypertrophic and class IIa HDACs being anti-hypertrophic (Zhang et al., 2002; Antos et al., 2003; Chang et al., 2004). Induced expression of HDAC2 in cardiomyocytes mimics hypertrophic growth in an Akt-dependent manner. In vivo, class I HDAC2 overexpressing transgenic animals develop cardiac hypertrophy whereas HDAC2-null animals are protected from cardiac hypertrophic response after stimulation either by pressure overload or isoproteranol (ISO) administration (Trivedi et al., 2007). Similar to HATs, HDACs also interact with DNA binding proteins regulating their activity. For instance, class IIa HDACs (HDAC4, -5, -7 and -9) can directly interact with MEF2 leading to inhibition of MEF2 activity and subsequent reduced cardiac hypertrophy (Backs and Olson, 2006). On the other hand, when MEF2 is discharged of HDACs, it may become an available target for HATs binding which in turn leads to enhanced activity of MEF2 (Backs and Olson, 2006).

**Table 1:** Role of HDACs in heart disease

Class	Chromatin modifying factor	Modulation	Phenotype	Mechanism
Class I	HDAC2	Germline deletion	Lethal at birth, Surviving adults are resistant to hypertrophy	Suppression of SRF and GATA4-dependent gene expression; Inhibition of hypertrophic Akt/GSK3β pathway
		Overexpression in myocardium	Cardiac hypertrophy	Activation of hypertrophic Akt/GSK3β pathway
		Deletion in myocardium	No cardiac phenotype	Redundancy with HDAC1
		Deletion of HDAC1 and HDAC2	Lethal at 2 weeks after birth: arrhythmias, dilated cardiomyopathy	Interaction with REST: repression of fetal genes involved in calcium handling and contractility
	HDAC3	Overexpression	Cardiac hyperplasia without hypertrophy	Suppression of Cdk inhibitors: promotion of cardiomyocyte proliferation
		Deletion in myocardium	Lethality at 3-4 months of age: cardiac hypertrophy, fibrosis, defects in fatty acid metabolism and lipid accumulation in the heart	Suppression of PPARα activity on gene promoters involved in metabolic regulation
Class II	HDAC5/HDAC9	Germline deletion	Enhanced hypertrophic response to cardiac stress; female hearts are protected from ischemia injury	Suppression of Mef2 and CAMTA2; suppression of Mef2-ERα-VEGFa pathway
Class III	SIRT1	Overexpression in myocardium	Low-moderate expression of SIRT1 reduces cardiac hypertrophy; High levels induces cardiac hypertrophy and apoptosis	SIRT1 expression is activated by cardiac stress and regulates the response to stress in a dose-dependent manner
	SIRT3	Germline deletion	Cardiac hypertrophy and fibrosis at 2 months of age	Inhibition the proapoptotic activity of Bax
		Overexpression in myocardium	Resistant to stress-induced cardiac hypertrophy	Activation of FOXO3a-dependent pathways; attenuation of the prohypertrophic MAPK/ERK and PI3K/Akt pathways.
	SIRT7	Germline deletion	Cardiac fibrosis, hypertrophy and shortened lifespan	Deacetlylation of p53; protection from stress-induced apoptosis

Besides transcriptional factors, HATs and HDACs can also interact with sarcomeric proteins. PCAF, a HAT, class II HDAC4 co-localizes with cardiomyocyte sarcomeres in the Z-disk whereas class I HDAC3 localize mainly in the A-band (Gupta et al., 2008; Samant et al., 2011). In addition, inhibition of HDAC4 results in altered calcium sensitivity and therefore altered contractility. HDAC4 has an unique docking site for the binding of calcium/calmodulin-dependent kinase II (CaMKII), which is absent in other class IIa HDACs. Phosphorylation of HDAC4 by CaMKII promotes nuclear export and derepression of HDAC target genes, which, in cardiomyocytes, will lead to hypertrophic growth (Backs et al., 2006), indicating a central role for CaMKII-HDAC4 signaling pathways in the development of cardiac hypertrophy. From the HDAC class IIb, HDAC6 catalytic activity seems to be consistently increased in stressed myocardium and is activated by different extracellular stimuli in cultured cardiac myocytes (Lemon et al., 2011). Recently, Cao et al. (2011) showed that inhibition of HDAC by TSA (HDAC inhibitor) treatment limits cardiac hypertrophy by suppressing autophagy. Further in vitro experiments, by selective downregulation of HDAC isoforms in cardiomyoctes, indicated HDAC1/2 as responsible for PE-induced autophagy (Cao et al., 2011). Autophagy is a self-degradative process during development and in response to nutrient stress, and can be altered under pathological conditions (Wang et al., 2013). Increasing evidence suggests more distinctive roles for HDACs besides only acting as histone deacetyltransferases.

#### Histone methylation

The most widely studied histone methylations are lysine methylations: histone H3 lysine 4 (H3K4), H3K9, H3K27, H3K36, H3K79 and H4K20 (Martin and Zhang, 2005). There is limited information about the function of histone methylation in HF. It is known that differential methylation patterns for H3K4 and H3K9 occur in the vicinity of various gene clusters of failing human hearts (Kaneda et al., 2009). Because such sets of genes encode proteins that function in the same signal transduction pathways and H3K9 mark-profile seems to be less sensitive to disease status, this indicates differential H3K4 marking during the development of HF (Kaneda et al., 2009). Furthermore, in a Dahl salt-sensitive rat model of congestive heart failure (CHF), genome-wide histone methylation analysis revealed H3K4me3 and H3K9me3 as the most abundant histone methylation marks (Kaneda et al., 2009). Interestingly, mapping of H3K4me3 and H3K9me3 enriched regions in the genome of human CHF samples compared to controls revealed many HF-associated genes located in these regions (Kaneda et al., 2009). Moreover, histone methylation has been shown to mark not only protein coding genes but also non-coding RNA regions (Movassagh et al., 2011a). The genome wide mapping of H3K36me3 in end-stage falling human hearts allowed to identify 4 novel non-coding RNA regions, which have active transcription and might be involved in HF (Movassagh et al., 2011a). This differential profile of histone methylation marks found in both human and animal samples suggests a potential role for HMTs and HDMs in HF. Accordingly, JMJD2A, a histone trimethyl demetyhlase (Kooistra and Helin, 2012), is found to be upregulated in human HCM patients compared to control (Zhang et al., 2011). Moreover, transgenic mice with cardiac-specific overexpression of JMJD2A develop exaggerated cardiac hypertrophy compared to control mice following transverse aortic constriction (TAC) whereas *jmjd2a*-null animals seem to be protected against TAC-induced cardiac stress (Zhang et al., 2011). All in all, these experiments indicate a potential modulator function for histone modification in HF.

## NON-CODING RNA IN HEART FAILURE

Post-transcriptional regulation of gene expression is mainly achieved by non-coding RNA molecules including miRNAs and, based on rather recent findings, long-noncoding RNAs (lncRNAs).

Comparison of miRNA expression profiles in healthy and failing heart samples from humans or animal models revealed differential miRNA expression patterns indicating their potential involvement in the development and progression of HF. In this regard, miRNA microarray analysis of cardiac tissue from mouse models of cardiac hypertrophy and CHF detected five upregulated miRNAs (namely miR-24, miR-125b, miR-195, miR-199a and miR-214), which were further confirmed in idiopathic end stage failing human hearts (van Rooij et al., 2006). Furthermore, mice overexpressing miR-195 developed pathological remodeling, impaired cardiac function and subsequently HF (van Rooij et al., 2006). Besides distinct expression signatures of miRNAs in healthy and failing hearts, the differential miRNA expression profile among failing hearts is dependent on the underlying HF etiology (Ikeda et al., 2007; Sucharov et al., 2008). Ikeda et al. (2007) found 14 aortic stenosis-specific miRNAs while a set of other eight miRNAs were mainly expressed in a cardiomyopathic form of HF. In a similar study, different sets of miRNAs were found for idiopathic dilated and ischemic cardiomyopathy (Sucharov et al., 2008). Furthermore, the expression levels of miRNAs can vary as the disease progresses (Bagnall et al., 2012). This was shown in a double transgenic mouse model, harboring mutations in both the myosin heavy chain gene and the cardiac troponin I gene, resulting in severe HCM and premature mortality by age of 21 days. Global miRNA profiles in those mice, at age of 10 and 16 days, revealed stable downregulation of specific miRNAs such as miR-1 and miR-133, suggesting a functional role for these miRNAs throughout the progression to HF. Counterwise, miR-31 was upregulated at the end-stage of HF which points to a specific function for this miRNA during the final phase of the disease (Bagnall et al., 2012).

Another miRNA microarray profiling study has been carried out in human end-stage CHF with or without left ventricular assist device (LVAD) compared to healthy subjects (Matkovich et al., 2009). Twenty-eight miRNAs were differentially expressed in diseased hearts regardless of LVAD support and, interestingly, the expression levels of 20 of those miRNAs were either normalized or reversed in the CHF group after LVAD support suggesting an eventual value of such miRNAs as prognostic tools for end-stage CHF patients (Matkovich et al., 2009). Recent data also emphasizes the variations between adult and child idiopathic dilated cardiomyopathy patients, regarding their miRNA expression profile (Stauffer et al., 2013). Naga Prasad et al. (2009) performed miRNA microarrays in end stage dilated cardiomyopathic hearts (with >15 ejection fraction) followed by in silico network analysis in order to obtain a global picture of the molecular networks and key proteins regulated by the dysregulated miRNAs. As a result, eight miRNAs displayed different expression levels in DCM subjects compared to controls and two out of these eight miRNAs, namely miR-7 and miR-378, were novel miRNAs, shown for the first time to be downregulated in end stage failing hearts (Naga Prasad et al., 2009). Confirmation of network analysis revealed upregulation of erythroblastic leukemia viral oncogene homolog 2 (ERBB2) and collagen, type I, alpha 1 (Col1A1) which are predicted targets of miR-7 and thus, confirming that the regulatory function of miRNAs results in alterations of global signaling networks during development and progression towards cardiac hypertrophy and HF (Naga Prasad et al., 2009).

A more recent study, besides showing that miRNA expression profiles differ between healthy and failing hearts, in consensus with previous findings, also demonstrated that failing adult hearts and fetal hearts display similar miRNA profiles supporting the paradigm of reactivation of a fetal gene program (Thum et al., 2007; Barry et al., 2008) at onset and/or during the development of HF.

On top of these profiling studies, a myriad of selected miRNAs were associated with cardiac disease-specific roles. miRNAs have also become a research focus on defining novel biomarkers of HF by characterizing miRNA patterns in easy accessible sources such as serum, plasma and even whole blood, and specific miRNA signatures have been identified as biomarkers of MI (Meder et al., 2011).

Interestingly, but not yet studied in the context of CVD, miRNA genes can be subject of DNA methylation with direct impact on the miRNA expression levels. Epigenetic-regulation of miRNA genes was mainly showed, so far, for different types of cancer. miRNAs such as miR-127 and miR-137 are sensitive to DNMT inhibitors and chromatin-modifying drugs. Interestingly, the miR-127 gene is embedded in a CpG island and is subject of epigenetic silencing (Saito et al., 2006). Because miR-127 is physiologically expressed as a member of a miRNA cluster together with miR-136, -431, -432 and -433 not only in normal tissues but also in cultured fibroblasts this could hint for a role of epigenetic regulation of this miR in cardiovascular disease, e.g., fibrosis, but this remains to be clarified (Saito et al., 2006). Similarly, the promoter region of miR-137 is heavily methylated in cancer cell lines and this is reversible after treatment with DNMT inhibitors (Balaguer et al., 2010).

To date, the epigenetic regulation of miRNA expression through methylation of CpG islands or other modifications in the promoter regions that encode for specific miRNAs has not been assessed in the context of CVD. Nevertheless, the above-mentioned studies strongly suggest a crucial role for such mechanisms at the onset of cardiovascular pathologies.

## PHARMACOEPIGENETICS IN HEART FAILURE

The existent therapies for HF seem to be insufficient since HF remains the leading cause of death in the developed countries. Therefore there is an increasing necessity for finding novel therapeutic targets. Because the wide variability in an individual𠆝s disease predisposition and response to treatment is only partially ascribed to heritable factors, epigenetical modifications diverging from DNA methylation to non-coding RNAs have gained much attention in several diseases, including HF (Feinberg, 2007; Mano, 2008). Therefore, epigenetic changes are currently being considered as therapeutic approaches in synergy with nucleotide variations at the drug response level (Szyf, 2004). This rapidly emerging new discipline, so-called pharmacoepigenomics, assesses the influence of epigenetic factors in the interindividual variation to drugs with the ultimate goal of discovering novel therapeutic targets (Ingelman-Sundberg et al., 2007). To date, the most advances have been made in the oncology field (Ingelman-Sundberg et al., 2007). However, the knowledge obtained from such studies combined with the knowledge on the role of epigenetic modifications is being applied to other complex forms of disease including HF.

In this context, several studies performed in animal models of disease endorse modifiers of epigenetic marks as therapeutic target points. Curcumin, a natural compound found in the spice trumeric, has an HAT inhibitory activity with specificity to p300/CREB-binding protein. It has been shown to rescue pathological cardiac remodeling and preserve cardiac function in two different rat models of HF, namely the salt-sensitive Dahl rats and in rats that were subjected to MI (Morimoto et al., 2008). An analogous study suggests that administration of curcumin in combination with a conventional therapy such as angiotension conversion enzyme inhibitors (ACEi), in MI-induced rats results in a beneficial additive effect on cardiac function (Sunagawa et al., 2011). Additionally, TSA, an HDAC inhibitor, was showed to blunt the hypertrophic response of cardiomyocytes to PE-treatment in a dose dependent manner and excluding the eventual cytotoxic effect of TSA (Antos et al., 2003). Moreover, treatment with TSA and valproic acid (VPA), another HDAC inhibitor, was able to attenuate cardiac hypertrophic growth in transgenic mice with cardiac overexpression of the atypical homeodomain protein Hop, known to be able to inhibit certain cardiac-gene expression by blocking serum response factor (SRP) transcription activity in a HDAC dependent way (Kook et al., 2003). TSA is also able to attenuate pathological cardiac remodeling in other mouse models such as isoprotenol-, angiotensin II- and pressure overload-induced hypertrophy (Kook et al., 2003; Kee et al., 2006).

Considering that epigenetics regulates phenotypic variation in health and disease, it is conceivable to expect that understanding and controlling the epigenome will prime great developments in the prevention and treatment of common diseases, including HF.

## CONCLUSION

The dynamic aspects of epigenetics may not only provide more accurate evidences to the role of changing environmental factors in the drug response, associating the environment with the genome, but also offer a way to reactivate silenced genes. While pharmacogenetics has been very valuable in the identification of interindividual variation in drug uptake and metabolism, epigenomics offers yet another layer of information that may help developing more personalized therapy. In the oncology field, epigenetic drugs have already entered the clinical arena and methylation patterns are used as biomarkers to subtype and stage various cancers as a critical and more personalized care (Coppede, 2011; Litzow, 2011).

It is clear that epigenetic modifications such as DNA methylation, histone modifications and RNA-based mechanisms are the molecular targets for disadvantageous environmental stimuli and may lead to the onset of other complex and heterogeneous diseases such as arrhythmia and HF. However, additional research is obviously necessary to further clarify how epigenetic mechanisms impact the onset and development of heart disease, to eventually identify new druggable targets in HF and allowing disease classification or risk stratification.

## Conflict of Interest Statement

The authors declare that the research was conducted in the absence of any commercial or financial relationships that could be construed as a potential conflict of interest.
